# Exome sequencing for assessing the risk of 453 monogenic disorders in offspring: A study of 832 Chinese couples

**DOI:** 10.1002/ctm2.70074

**Published:** 2024-10-25

**Authors:** Xulong Ding, Miao Jiang, Qin Hu, Ruiqing Tong, Lin Wang, Jinxing Lv, Ling Pan, Jianquan Hou, Jun He, Peng Zhou

**Affiliations:** ^1^ Center of Translational Medicine and Clinical Laboratory, The Fourth Affiliated Hospital of Soochow University, Medical Center of Soochow University, Suzhou Dushu Lake Hospital Suzhou Jiangsu China; ^2^ Department of Cardiology The Fourth Affiliated Hospital of Soochow University, Medical Center of Soochow University Suzhou China; ^3^ Suzhou Basecare Medical Laboratory Co., Ltd Suzhou Jiangsu China; ^4^ Reproductive Medicine Center, The Fourth Affiliated Hospital of Soochow University, Medical Center of Soochow University, Suzhou Dushu Lake Hospital Suzhou Jiangsu China; ^5^ Department of Urology The Fourth Affiliated Hospital of Soochow University Suzhou China; ^6^ HLA Laboratory of Jiangsu Institute of Hematology, Collaborative Innovation Center of Hematology, The First Affiliated Hospital of Soochow University, 13/F (West), Hospital Comprehensive Building Suzhou Jiangsu China

Dear Editor,

Birth defects are abnormalities that occur during intrauterine life,^1^, ^2^, ^3^, ^4^ in particular, monogenic disorders stand out as a substantial contributor to birth defects,^5^ constituting approximately 22.2% of all birth defects.^6^ Due to the lack of evident abnormalities during fetal development in most autosomal recessive and X‐linked genetic disorders, identification of recessive monogenic disorders often occurs only after the birth of an affected child.^7^ Early screening and diagnosis play a crucial role in the control of these diseases and have important scientific and social significance. In China, the incidence of birth defects is approximately 5.6%,^8^ current routine newborn screening in most parts of China is still limited and specific and includes screening for four genetic metabolic diseases (phenylketonuria, congenital hypothyroidism, congenital adrenal hyperplasia and galactosemia) and hearing disorders, and we has not yet established a comprehensive system for the prevention and control of birth defects caused by other monogenic disorders. Here, we developed a detection system based on whole exome sequencing (ES) that includes 453 monogenic disorders with high prevalence in the Chinese population and the associated genetic variants. This system was applied to test 832 couples, followed by a 2‐year follow‐up. We identified genes with higher variant frequencies for individuals and couples, as well as cases of birth defects identified through ES results during follow‐up. These findings further underscore the importance of ES in assessing the risk of monogenic disorders in offspring, enabling informed reproductive decisions.

The overall study design was described in Figure S1. A total of 832 couples were screened for eligibility for inclusion between December 2021 and 3 December 2022. The participant demographics of the cohort were listed in Table S1. The mean (± SD) age was 29.29 ± 3.29 years for females and 30.27 ± 3.60 years for males. Most couples had either not had offspring (36.5%, 304/832) or were still pregnant (6.0%, 50/832), with only 59 (7.1%, 59/832) couples having a reproductive history of one or more pregnancies. Furthermore, there was a significant number of couples who experienced miscarriages (14.4%, 120/832).

According to the carrier rates in the Chinese and Asian populations,^9^ we included 453 types of monogenic disorders (Table S2), the classification and inheritance patterns were shown in Figure 1A and Table S2. Subsequently, we analysed the ES results and categorised mutations related to monogenic disorders in 832 couples, as depicted in Figure 1B. There were no significant differences in the proportions of the six categories between males and females, with pathogenic [P] constituting the largest proportion (female: 46.2%; male: 46.9%). Next, we classified all individuals into non‐carrier, carriers (heterozygous) and carriers (homozygous) as described in methods in Supplementary Material, with 3 females and 2 male in homozygous carriers’ group, 660 females and 634 male in heterozygous carriers’ group and 169 females and 196 male in non‐carriers’ group(Figure 1C, Table S3). In the carriers’ group, the gap junction beta‐2 protein (*GJB2*) gene had the highest frequency of P or LP variants (Figure S2), occurring in approximately 17.4% (226/1299) of the individuals in the abnormal group. Other genes with higher variant frequencies in this population included cytochrome P450 family 21 subfamily A member 2 (*CYP21A2*), cystic fibrosis transmembrane conductance regulator (*CFTR*), and UDP glucuronosyltransferase family 1 member A1 (*UGT1A1*). We also conducted a statistical analysis of the frequency of the top 10 P or LP variants in this cohort and compared it with the Genome Aggregation Database (gnomAD)^10^; the results were shown in Table 1. We found that the p.Val37Ile variant in *GJB2* had the greatest frequency in this cohort (overall: .050; male: .046; female: .053), exceeding the variant rate in the overall population database (.050 vs. .0077) and slightly lower than that in the East Asian population (.050 vs. .083). Notably, we identified some sites with low variant frequencies in the database but higher variant frequencies in our population. For example, the frequency of p.Pro229Gln in *UGT1A1* was .012 in our cohort but only .0019 in gnomAD. Detailed information on the gene and protein alterations in the cohort was provided in Table S3. Next, couples were classified into the positive diagnosis group and the negative diagnosis group as described in methods, and 71 couples were identified as having a positive diagnosis, for an overall diagnostic rate of 8.5% (71/832). These 71 couples exhibited three positive patterns as mentioned in Supplementary Material (Table S3), with 21 couples in which both individuals had the same P or LP variants (Pattern 1), 15 couples with P or LP variants that conformed to the disease pattern (Pattern 2), and 35 couples in which one individual had P or LP variants and the other had VUSs in the same gene (Pattern 3) (Figure 1D). Detailed information on the number of P or LP variants for each positive pattern was provided in Tables 2 and S3. Based on these data, we compiled the top four genes with variants and their corresponding positive patterns, as shown in Figure 1E. Consistent with the individual data, variants in *GJB2* also occurred most frequently in couples classified as positive.

**FIGURE 1:**
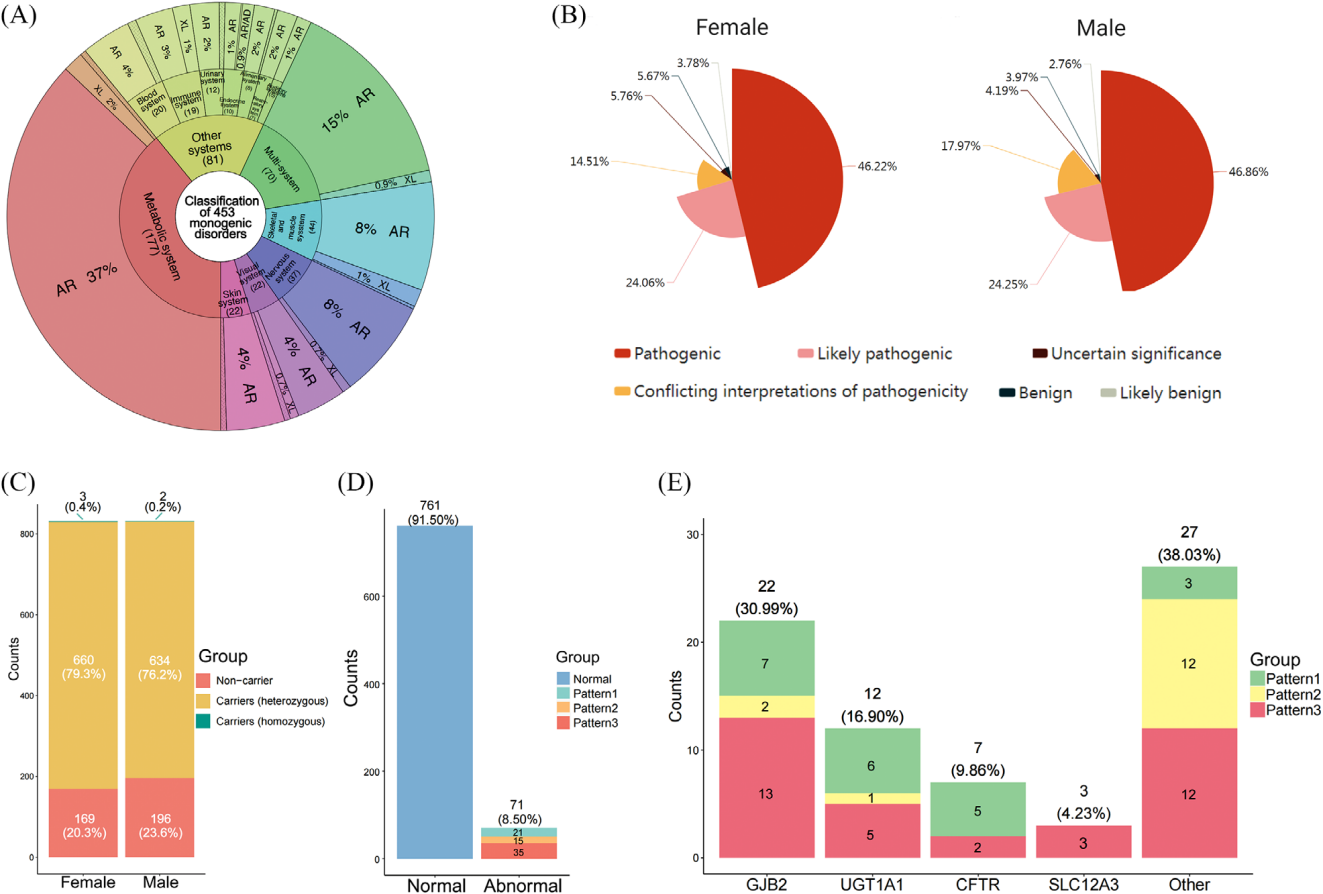
The diagnostic rate and associated gene variants in the abnormal population and positive couples. (A) The classification and inheritance patterns of 453 monogenic disorders. (B) The proportion of pathogenicity of genetic mutation sites for female and male. (C) The number of non‐carrier, carriers (heterozygous) and carriers (homozygous) between males and females. (D) The number of positive diagnostic couples and their positive pattern. (E) Top four genes with variants and their corresponding positive patterns among positive diagnostic couples. AR: autosomal recessive; AD: autosomal dominant; XL: X‐linked dominant/recessive; Pattern 1: Male and female with the same pathogenic (P) or likely pathogenic (LP) variants; Pattern 2: male or female with P or LP variants that conform to the disease pattern; Pattern 3: male or female with a P or LP variant and the other with variants of uncertain significance (VUSs) in the same gene.

**TABLE 1:** Top 10 genetic variants with the highest frequency in the cohort.

Gene	RefSeq identifier	HGVS consequence	Group	Count	Number	Frequency	Allele Frequency (Total)^a^	Allele Frequency (East Asian)^a^
GJB2	NM_004004.6	p.Val37Ile	Overall	165	3328	.050	.0077	.083
			Female	88	1664	.053		
			Male	77	1664	.046		
		p.Leu79Cysfs*3	Overall	41	3328	.012	.00047	.0062
			Female	14	1664	.0084		
			Male	27	1664	.016		
CYP21A2	NM_000500.9	p.Gln319Ter	Overall	147	3328	.044	.0001	/^b^
			Female	70	1664	.042		
			Male	77	1664	.046		
UGT1A1	NM_000463.3	p.Pro364Leu	Overall	71	3328	.021	.0012	/^b^
			Female	36	1664	.022		
			Male	35	1664	.021		
		p.Pro229Gln	Overall	41	3328	.012	.0015	.0019
			Female	24	1664	.014		
			Male	17	1664	.010		
SMPD1	NM_000543.5	p.Lys189Glnfs*4	Overall	49	3328	.015	.0002	/^b^
			Female	26	1664	.016		
			Male	23	1664	.014		
GALC	NM_000153.4	p.Leu634Ser	Overall	42	3328	.013	.00065	.0085
			Female	21	1664	.013		
			Male	21	1664	.013		
POLG	NM_002693.3	p.Arg964Cys	Overall	34	3328	.010	.00068	.0088
			Female	12	1664	.0072		
			Male	22	1664	.013		
SLC12A3	NM_001126108.2	p.Val578Met	Overall	20	3328	.0060	.00065	.0083
			Female	7	1664	.0042		
			Male	13	1664	.0078		
MLC1	NM_015166.3	p.Arg22Gln	Overall	19	3328	.0057	.00047	.0044
			Female	12	1664	.0072		
			Male	7	1664	.0042		

Abbreviations: *CYP21A2*, cytochrome P450 family 21 subfamily A member 2; *GALC*, Galactosylceramidase; *GJB2*, gap junction beta‐2 protein; *MLC1*, Modulator Of VRAC Current 1; *POLG*, DNA polymerase subunit gamma; *SLC12A3*, Solute carrier family 12 member 3; *SMPD1*, Sphingomyelin Phosphodiesterase 1; *UGT1A1*, UDP glucuronosyltransferase family 1 member A1.

^a^ Data from the Genome Aggregation Database (gnomAD);.

^b^ ‘/’ represents no record in gnomAD.

**TABLE 2:** Top 10 genetic variants of positive couples.

	HGVS consequence	Counts
**Pattern 1** ^a^		
UGT1A1	p.Pro364Leu	6
GJB2	p.Val37Ile	5
CFTR	c.1210‐12T[5]/c.1210‐34TG[12]	4
Other	/	6
**Pattern 2** ^b^		
*Autosomal dominant*		
ALPL	p.Ser181Leu	2
	p.Ala116Thr	1
F11	p.Leu483Phefs*2	1
	p.Gly418Val	1
	c.55+2T > C	1
ABCC8	p.Arg1352His	1
	p.Asp1471Asn	1
Other	/	4
*Autosomal recessive*		
GJB2	p.Val37Ile	2
BCHE	p.Gln147Ter	1
**Pattern 3** ^c^		
GJB2	/	13
UGT1A1	/	5
SLC12A3	/	3
ABCA4	/	2
ABCG5	/	2
Other	/	10

Abbreviations: *ABCA4*, ATP binding cassette subfamily A member 4; *ABCC8*, ATP Binding Cassette Subfamily C Member 8; *ABCG5*, ATP Binding Cassette Subfamily G Member 5; *ALPL*, alkaline phosphatase, biomineralisation associated; *BCHE*, butyrylcholinesterase; *CFTR*, cystic fibrosis transmembrane conductance regulator; *F11*, coagulation factor XI; *GJB2*, gap junction beta‐2 protein; *SLC12A3*, Solute carrier family 12 member 3; *UGT1A1*, UDP glucuronosyltransferase family 1 member A1.

^a^ Pattern 1: Male and female with the same pathogenic (P) or likely pathogenic (LP) variants.

^b^ Pattern 2: Male or female with P or LP variants that conform to the disease pattern.

^c^ Pattern 3: Male or female with a P or LP variant and the other with variants of uncertain significance (VUSs) in the same gene.

To further confirm the diagnostic value of ES, we conducted a 2‐year follow‐up investigation of 832 couples, and clinical outcomes were available in Tables 3 and S4. A total of 214 couples had children, and most of the children were healthy at birth. However, 6 children were diagnosed with genetic disorders either at birth or shortly afterward, with 4 of them born to couples with positive results. This finding implies that the risk of giving birth to unhealthy children was significantly greater for couples with positive results than for couples with negative results (odds ratio 19.3, *p *< .001). In the same period, 320 couples had not had children for various reasons, including no plans to have children, pregnancy in progress, miscarriages, and infertility. Similarly, couples with positive results had a greater risk of miscarriage and infertility than did couples with negative results (odds ratio 2.26, *p *= .049).

**TABLE 3:** Clinical outcomes of negative and positive couples during 2 years of follow‐up.

	Cohort, No. (%); mean (SD)^a^		
	Negative couples	Positive couples	*p* value^b^
Maximum, *n*	761	71	/
**Fetal birth**			
Healthy	189 (24.8%)	19 (26.8%)	< .001
Unhealthy	2 (.3%)	4 (5.6%)	
**No offspring**			
No plan	125 (16.4%)	10 (14.1%)	.049
In pregnancy	87 (11.4%)	6 (8.5%)	
Miscarriage	19 (2.5%)	5 (7.0%)	
Infertility	58 (7.6%)	10 (14.1%)	
**Missing**	281 (37.0%)	17 (23.9%)	

Abbreviation: *SD*, standard deviation.

^a^ Frequencies and percentages reported for categorical variables, means and standard deviations reported for continuous variables, unless otherwise noted.

^b^ *p* Value was obtained from the chi‐square test or Fisher's exact test.

Furthermore, with the informed consent of the families, we conducted a more in‐depth study on 2 families who exhibited ultrasound abnormalities during pregnancy. Family 877 entered the cohort at 20 weeks of pregnancy and underwent ES (Figure 2A). The ES results revealed the same heterozygous variant in polycystic kidney and hepatic disease 1 (*PKHD1*) (c.11314C > T) in both individuals, and Sanger sequencing was performed to confirm this finding. According to the ClinVar and HGMD databases, this variant was confirmed to be pathogenic, and this variation leads to polycystic kidney disease 4 with or without polycystic liver disease (PKD4), a severe autosomal recessive monogenic inherited disorder primarily affecting the kidneys and liver in children. At 28 weeks of pregnancy, the woman had abnormal ultrasound results indicating clinical manifestations of the disease – enlarged fetal kidneys and abnormal cortical echoes (Figure 2B). Five weeks later, after consultation with the patient, the decision was made to induce labour. To confirm the gene variant, we conducted ES using the umbilical cord and cord blood. The results revealed a homozygous variant in *PKHD1* (c.11314C > T) in the neonate (Figure 2C), and Sanger sequencing was performed to confirm the variant (Figure 2D). Unfortunately, the fetus passed away two weeks later.

**FIGURE 2:**
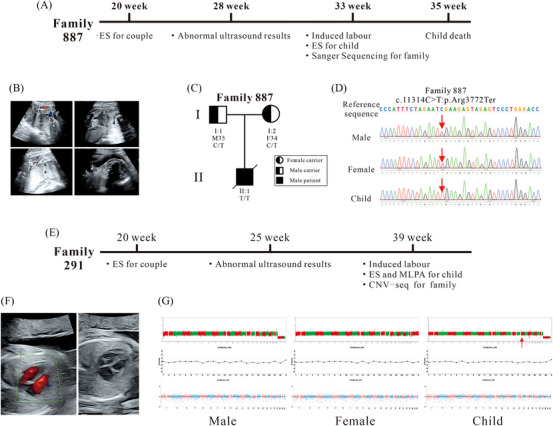
The in‐depth studies on Family 877 and Family 291. (A) Diagrammatic drawing of the pregnancy and subsequent testing results for Family 887. (B) Abnormal ultrasound results for family 887 at 28 weeks of pregnancy. (C) The pedigree plot of family 887 with genotypes for c.11314C > T, p.Arg3772Ter, PKHD1 and phenotypes. (D) Sanger sequencing results for the *PKHD1* gene. The red arrow indicates the variant in *PKHD1* (c.11314C > T). (E) Diagrammatic drawing of the pregnancy and subsequent testing results of Family 291. (F) The abnormal ultrasound results of Family 291 at 25 weeks of pregnancy. (G) The CNV results of Family 291 and their child. The red arrow indicates the deletion in the q12 region of chromosome 17.

Family 291 was enrolled at 21 weeks of pregnancy and underwent ES, which revealed no P/LP variants in the couple (Figure 2E). However, at 25 weeks of pregnancy, the woman presented with abnormal ultrasound results indicating fetal bilateral renal enlargement and abnormal cortical echoes, symptoms closely resembling those of polycystic kidney disease (Figure 2F). After 14 weeks, following consultation with the patient, the decision was made to induce labour. Simultaneously, ES was performed on the fetal umbilical cord and cord blood to determine whether the fetus had polycystic kidney disease. The results showed no clinically significant variants related or partially related to the clinical information provided by the patient. Additionally, no exon copy number abnormalities were detected in polycystin 1, transient receptor potential channel interacting (*PKD1*), or polycystin 2, transient receptor potential cation channel (*PKD2*) by MLPA (Figure S3). To further investigate the cause of the fetal ultrasound abnormalities, CNV‐seq analysis of the ES data was conducted using bioinformatics methods, and the results suggested a suspected heterozygous deletion in the chr17:34493374‐36387115 region. Subsequently, CNV‐seq was employed to confirm this finding, ultimately revealing a 1.73 Mb deletion in the q12 region of chromosome 17 (seq[GRCh37/hg19] 17q12 (34470001‐36520000)×1) (Figure 2G). This deletion will cause renal cysts and diabetes syndrome due to haploinsufficiency of HNF1B. Renal cysts and diabetes syndrome is an autosomal dominant disorder characterised by developmental kidney abnormalities. This finding likely explains the cause of the fetal ultrasound abnormalities. Therefore, guided by the depth analysis of ES results and CNV‐seq, we successfully identified the etiology.

In conclusion, our study established a population cohort comprising 1664 participants and performed ES. The analysis of the ES results provided gene mutation information for approximately 453 monogenic disorders, which fills the gap in the frequency of P/LP variant sites in the Chinese population. Our 2‐year follow‐up results revealed a correlation between ES results and miscarriages, as well as double implications for gene mutations and chromosomal abnormalities in ES results. Combining genomic data with corresponding clinical data, our study demonstrated that carrier screening based on ES serves as an important method for reducing birth defects, emphasising its necessity for pre‐pregnancy screening in China.

## AUTHOR CONTRIBUTIONS

*Conceptualisation*: Jun He, Peng Zhou, Jianquan Hou, Miao Jiang; *Funding acquisition*: Xulong Ding, Ruiqing Tong; *Data curation*: Xulong Ding; *Investigation*: Ling Pan, Ruiqing Tong, Lin Wang, Jinxing Lv; *Methodology*: Qin Hu, Ling Pan; *Writing – original draft*: Xulong Ding; *Writing – review & editing*: Xulong Ding, Jun He.

## CONFLICT OF INTEREST STATEMENT

The authors declare that they have no competing interests.

## FUNDING

This work was supported by National Natural Science Foundation of China (82301362); Natural Science Foundation of Jiangsu Province (BK20230277); Natural Science Foundation of Jiangsu Higher Education Institutions of China (23KJB180023); Suzhou Science and Technology Bureau project (SZM2021016, SZM2023037), the Gusu Talent Program (2023‐055) and Jiangsu Provincial Double‐Innovation Doctor Program (JSSCBS20230499).

## ETHICS STATEMENT

This study was approved by Ethics Committee in The Fourth Affiliated Hospital of Soochow University (identifier: 210095), and written informed consent was obtained from all included couples for genetic test. This study was performed in accordance with the principles of the Helsinki Declaration.

## CONSENT FOR PUBLICATION

All authors have agreed to the submission and publication of this study.

## Supporting information



Supporting information

Supporting information

Supporting information

## Data Availability

The datasets supporting the major results/conclusions of this article are included within the article and its additional files. Our sequencing raw data cannot be submitted to publicly available databases because the ethical approval did not permit sharing of exome sequencing data and the patients’ families did not consent to share their raw data.
