# Supplementary material for: Exome sequencing for assessing the risk of 453 monogenic disorders in offspring: A study of 832 Chinese couples
Source: Clin Transl Med. 2024 Oct 25;14(11):e70074. doi: 10.1002/ctm2.70074 (PMC11511670; doi:10.1002/ctm2.70074)
Supplement: Supplementary file 1 — Supporting information [file CTM2-14-e70074-s001.docx]

Supplemental material (Methods) for

**Exome Sequencing for Assessing the Risk of 453 Monogenic Disorders in Offspring: A Study of 832 Chinese Couples**

Xulong Ding^1, a, *^, Miao Jiang^2, a^, Qin Hu^3, a^, Ruiqing Tong^4^, Lin Wang^1^, Jinxing Lv^4^, Ling Pan^3^, Jianquan Hou^5^, Jun He^1, 6, *^, Peng Zhou^1, *^

^1^ Center of Translational Medicine and Clinical Laboratory, The Fourth Affiliated Hospital of Soochow University, Medical Center of Soochow University, Suzhou Dushu Lake Hospital, Suzhou, Jiangsu 215123, China.

^2^ Department of Cardiology, The Fourth Affiliated Hospital of Soochow University, Medical

Center of Soochow University, Suzhou, China.

^3^ Suzhou Basecare Medical Laboratory Co., LTD, Suzhou, Jiangsu 215123, China.

^4^ Reproductive Medicine Center, The Fourth Affiliated Hospital of Soochow University, Medical Center of Soochow University, Suzhou Dushu Lake Hospital, Suzhou, Jiangsu 215123, China.

^5^ Department of Urology, The Fourth Affiliated Hospital of Soochow University, Suzhou 215000,

China.

^6^ HLA Laboratory of Jiangsu Institute of Hematology, Collaborative Innovation Center of

Hematology, The First Affiliated Hospital of Soochow University, 13/F (West), Hospital

Comprehensive Building, No.899 Ping Hai Road, Suzhou 215031, Jiangsu, China.

1. **Study Design and Participants**

This study was approved by the Ethics Committee of The Fourth Affiliated Hospital of Soochow University (identifier: 210095), and written informed consent was obtained from all couples undergoing genetic testing. The study cohort was recruited between December 28, 2021, and December 3, 2022. A total of 2466 registered participants (1233 couples) were screened for eligibility for inclusion according to the following criteria: (1) couples in the preconception and early pregnancy period with the intention of reproduction; (2) individuals whose offspring exhibited normal phenotypes but wanted to understand their reproductive risks and were concerned about the health of their offspring; (3) couples experiencing infertility; (4) individuals with a family history of genetic disorders; (5) individuals in consanguineous marriages; (6) populations at high risk for genetic disorders due to specific ethnic or regional backgrounds; (7) individuals utilizing assisted reproductive technologies to conceive; (8) couples with blood samples available for both individuals; and (9) couples for whom the quality of the DNA sample was sufficient for ES. In total, 1664 participants (832 couples) were included and successfully underwent ES.

1. **Exome sequencing, analysis and interpretation**

All genomic DNA was extracted from peripheral blood for couples, and fetal DNA was obtained from umbilical cord and cord blood. Exome Library Construction Kit (BASECARE) was used for DNA library prepared, followed by sequenced using MGISEQ-2000 following the manufacturer’s instructions.

Raw fastq reads were filtered by using Trimmomatic[1] (v0.36) to remove low quality and adapter contaminated reads, and mapping of clean read sequences was performed with human reference genome (hg19) by using Burrows Wheeler Aligner (BWA) [2] (v0.7.17) algorithm. Genome Analysis ToolKit [3] (GATK4) was employed for discarding duplicate sequences, local indel realignment, base quality recalibration and haplotypecaller variant calling. Variant annotation was conducted with Annovar [4] (v2017Jul17/v2020Apr01) and InterVar[5](v2022June). Allele frequency information from 1000 Genome Project (1000G Phase 3 v5a), Genome Aggregation Database (gnomAD r2.1/v2.1.1) and Exome Aggregation Consortium (ExAC r0.3.1) were annotated. Multiple software such as SIFT [6], Polyphen2 [7], MutationTaster [8], MutationAssessor [9], Provean [10], CADD [11] and REVEL [12] were applied for protein function prediction. Gene/variants were additionally annotated according to ClinVar, the professional version of the Human Gene Mutation Database (HGMD professional v2018.2 & v2021.2) and intervardb. Besides, CNV analysis were performed by using DECoN[13] (v1.0.1) and annotating and filtering identified CNVs using custom-built software. Quality control for each sample included raw reads >= 100,000,000, raw bases >= 15G, Q30 >= 80%, an average depth of > 100X and > 95% targeted region with at least 20X.

According to the standards and guidelines published by the ACMG and the carrier rates in the Chinese and Asian populations[25], we ultimately analyzed 453 genetic diseases across various categories, including metabolic system, skeletal and muscle system, nervous system, visual system, skin system and other systems, including three inheritance patterns (AR: Autosomal recessive; AD: Autosomal dominant; XL: X-linked dominant/recessive). (**Additional File 2, Table S2**). All the single-nucleotide variants (SNVs) and insertions/deletions (indels) detected were classified into six categories (pathogenic [P], likely pathogenic [LP], conflicting interpretations of pathogenicity, uncertain significance [VUS], likely benign [LB], and benign [B]).

ES results for individuals were classified into two tiers: (1) **Non-carrier**: P or LP variant sites were not identified in 453 pathogenic genes; (2) **Carriers (heterozygous)**: Individuals who carry one normal allele and one mutated allele for 453 genetic diseases; (3) **Carriers (homozygous)**: Individuals who carry a specific genetic mutation for 453 genetic diseases, with both alleles being mutated

For couples, a positive diagnostic result encompassed the following patterns: **Pattern 1**: male and female with the same P or LP variant site; **Pattern 2**: male or female with a P or LP variant site that conformed to the disease pattern; **Pattern 3**: male or female with a P or LP variant site, and the other with a VUS in the same gene; and Pattern 4: female with X-linked P, LP, or VUSs. All other results were considered negative diagnostic results.

**3. Copy Number Variation (CNV) sequencing, variant detection and annotation**

Blood sample pre-processing and DNA library prepared methods were referenced from WES, followed by sequenced using DA8600 following the manufacturer’s instructions.

Raw fastq reads were mapped with human reference genome (hg19) by using TMAP (5.6.8), and BamDuplicates (5.12.27)、Samtools (1.2) and bedtools were employed for discarding duplicate sequences from PCR and genome, along with low quality reads. After dividing the chromosome into non-overlapping 20kb fragment size by CHD_NT_UR (1.0), CNV-seq (1.0) was performed for GC calibration, Copy number calculation.

Quality control for each sample included raw reads >= 5.0Mb, Unique reads >= 3.5 Mb, coverage of reference genome >= 10% and the mapping rate >= 98%.

1. **Multiplex ligation-dependent probe amplification (MLPA)** **analyses**

All genomic DNA was extracted from peripheral blood for couples, and fetal DNA was obtained from umbilical cord and cord blood. MLPA analyses were performed with SALSA MLPA Probemix P352 PKD1-PKD2 (MRC-Holland) following the manufacturer’s instructions.

The genomic DNA is denatured to separate the double-stranded DNA into single strands. MLPA probes are added to the denatured DNA. Ligase enzyme is introduced, and PCR (Polymerase Chain Reaction) is employed to selectively amplify the ligated probes. The PCR products were analyzed by capillary electrophoresis using a 3730 DNA analyzer. (Applied Biosystems). The sizes and peak values of the PCR products were analyzed using the GeneMapper software V4.0 (Applied Biosystems). The sample data were analyzed using the Coffalyser software (MRC-Holland). According to the data provided in the instruction manual for the SALSA MLPA kit, the results for normal exon, exon deletions, exon duplications, suspected exon deletions, and suspected exon duplications are 0.7–1.3, 0–0.3, 1.7–2.3, 0.3–0.7, and 1.3–1.7, respectively. The probe sequence is provided in the table below.

**Table.** The probe sequence of SALSA MLPA Probemix P352 PKD1-PKD2

| **Gene** | **Mapview** | **LPO** | **RPO** |
| --- | --- | --- | --- |
| CASP2 | 07-142,710996 | CAGATGAGACTGATCGTGGGGTTGACCA | ACAAGATGGAAAGAACCACGCAGGATCCCCT |
| KLK3 | 19-056,050009 | GAGAGCTGTGTCACCATGTGGGTCCCG | GTTGTCTTCCTCACCCTGTCCGTGACGTGGA |
| IL4 | 05-132,037606 | TTGCCTCACATTGTCACTGCAAATCGACACCTAT | TAATGGGTCTCACCTCCCAACTGCTTCCCCCT |
| LMNA | 01-154,371238 | CGCCGTCATGAGACCCGACTGGTGGAGATT | GACAATGGGAAGCAGCGTGAGTTTGAGAGCCGGCT |
| ATP7B | 13-051,422428 | CGTCAAGGTGGTCCCTGGGGGAAAGTT | TCCAGTGGATGGGAAAGTCCTGGAAGGCAATACCATGGCT |
| ARHGEF10 | 08-001,812192 | GCTCCTTGGAATACGGATGGAGTTCGAGTGAAT | TTGAAAGTTACGAAGAGCAGAGTGACTCGGAGTGCAAGAATGG |
| IGF1R | 15-097,300054 | CAGAGACCTTGCTGCCCGGAATTGCATGGTAGCCGA | AGATTTCACAGTCAAAATCGGAGGTGTGTCCTTAGCTTTCCAGGTC |
| PAX3 | 02-222,868507 | GGCATGTTCAGCTGGGAAATCCGAGACAA | ATTACTCAAGGACGCGGTCTGTGATCGA |
| PKD1 | 16-002,101496 | GTGCGCGTCTTTGAGGAGCTCCGCGGA | CTCAGCGTGGACATGAGCCTGGCCGTGGAGC |
| PKD1 | 16-002,096168 | GTTCTGTGTCTACAAGGGCAGCCTCTCCA | GCTACGGAGCCGTGCTGCCCCCGGGTTT |
| PKD1 | 16-002,095482 | CTGCTGGCCTCCTGCCGGCCTCCTGCG | CTGCTGACAGCTTGCTGTGCCCCCTGCCTGC |
| PKD1 | 16-002,087302 | CCAGCCCCTACTCGCCTGCCAAATCCTTC | TCAGCATCAGGTGAGCTGGGGTGAGAGGA |
| PKD1 | 16-002,083964 | CCAGGACTGGTGGAGGGTCTGCGGA | AGCGCCTGCTGCCGGCCTGGTGTGCCTCCCT |
| PKD2 | 04-089,148048 | TGCGAGCAGCGGGGCCTGGAGATCGAGAT | GCAGCGCATCCGGCAGGCGGCCGCGCGGG |
| PKD2 | 04-089,159646 | CAAGACTCATGGAGGAAAGCAGCACTAACCGA | GAGAAATACCTTAAAAGTGTTTTACGGGAACTGGT |
| PKD2 | 04-089,178433 | GAAGGCTCCTTATTGGATGGGCTGTACTGGA | AGATGCAGCCCAGCAACCAGACTGAA |
| PKD2 | 04-089,183468 | CTTATAGTGGAGCTGGCTATTATCTGGATTTGTCAA | GAACAAGAGAGGAAACAGCTGCACAAGTT |
| PKD2 | 04-089,192204 | CATCAAATGTGGAGGTGCTACTACAGTTTCTGGAAG | ATCAAAATACTTTCCCCAACTTTGAGCATCTG |
| PKD2 | 04-089,198186 | CGATATCAACTTTGCAGAGATTGAGGAAGCTAATCG | AGTTTTGGGACCAATTTATTTCACTACATTTGTGTTC |
| PKD2 | 04-089,202105 | TGATACTTACTCTGAAGTGAAATCTGACTTGGCAC | AGCAGAAAGCTGAAATGGAACTCTCAGATCT |
| PKD2 | 04-089,205610 | GAGAGTCTGCGGCAAGGAGGAGGCAA | GTTAAACTTTGACGAACTTCGACAAGATCTCAAA |
| PKD2 | 04-089,205966 | GCAATATTCACAAAGTACGACCAAGATGGAGACCAA | GAACTGACCGAACATGAACATCAGCAGAT |
| PKD2 | 04-089,215710 | GGGAATCCGATGATGCAGCTTCCCAGA | TCAGTCATGGTTTAGGCACGCCAGTGGGACT |
| PKD1 | 16-002,092014 | GGGCCGGGGCTCAGGTGAGGGGCGCA | GCGGGGTGGCAGGGCCTCCCCTGCTCTCACTGGCTGT |
| PKD1 | 16-002,087832 | ACGTTCTCAGGCCTCCACGCTGAGGTGAGGA | CTCTACTGGGGGTCCTGGGCTGGGCTGGGGG |
| PKD1 | 16-002,083564 | CGTACGGCCACCCCACGGCTTTGCACTCTTCCT | GGCCAAGGAAGAAGCCCGCAAGGTCAAGAGG |
| PKD1 | 16-002,094543 | GCAGACGCTGCACAAGCTGGAGGCCATGATGCT | CATCCTGCAGGCAGAGACCACCGCGGGCACC |
| PKD1 | 16-002,106815 | CTCAGCGCCGCACAGCTACGTCTGCGAGCTGCA | GCCCGGAGGTGTGCGGGGGGCCAGGCAG |
| PKD1 | 16-002,127382 | TTATTCCACAACCGCCTTCAAACAATCATTGAGACTTG | GTTAATCTGTTTTGCTCATTTGGCAGCAGTTTCTTGTGGCTGTTTC |
| PKD1 | 16-002,127880 | TTATCAAGAAACATTTCCCTCACGTCTTCTTCCCTGAAC | CAAACAAGATCTCTGGCACATTTTATTTGCTCTGTCTCACCACATGG |
| PKD2 | 04-089,186871 | AGCCTTTAAAGCTGATCCGATATGTCACAACTTT | TGATTTCTTCCTGGCAGCCTGTGAGATTATCTTTTGTTTCTTT |
| PKD2 | 04-089,186954 | TACTATGTGGTGGAAGAGATATTGGAAATTCGCATTCACAAA | CTACACTATTTCAGGAGTTTCTGGAATTGTCTGGATGTTGTGATCG |
| PKD2 | 04-089,147894 | GACGCCAGTGACCGCGATGGTGAACTCCAGT | CGCGTGCAGCCTCAGCAGCCCGGGGACGCCAAGCG |
| PKD2 | 04-089,159708 | CTGGTCACATACCTCCTTTTTCTCATAGTCTTGTGCATCTGTA | AGTAGAATATTTCCTTGCACTAATGGGAAAGTTTTGAAAAGAT |
| NF1 | 17-026,708406 | TTGGCAGGCTACACTGGTAAAATATACCACAGATGAGTTTGATCAAC | GAATTCTTTATGAATACTTAGCAGAGGCCAGTGTTGTGTTTCCCAAAGT |
| ERG | 21-038,869457 | GAACAGCTGGTAGATGGGCTGGCTTACTGAAGG | ACATGATTCAGACTGTCCCGGACCCAGCAGCTCATATCAA |
| PKD1 | 16-002,107535 | GCAGGCGCAGGAGCAGTGTCA | GGCCTGGGCCGGGGCCGCCCTGGCAATGGTG |
| KCNIP4 | 04-020,343401 | ACGATATGATGGGTAAATGTACATATCCTGTCCTCAA | AGAAGATGCTCCCAGACAACACGTTGAAACATTTTTTCAGG |
| PKD2 | 04-089,196285 | CAGGACCATGAGCCAGCTCTCGA | CAACCATGTCTCGATGTGCCAAAGACCTG |
| PKD2 | 04-089,208145 | CGCCACTAGAAATGCTTCCCCTCCTTCTGG | AGCTATGTCCGCTATCTTCATCGTCATCCTCCTCAGAGTCA |
| PKD1 | 16-002,109492 | GAGCTCCCCACTCCCAGAGGTCAGGAGGGGA | CTTTCTGATGGAAGACCCAAATGAACACTCATCTG |
| PKD1 | 16-002,092826 | TGCTCGGCTAGCAGGAGGATCCGCCCAGAGTCA | CTCCAGGGTGCTGACCACCGGCCCTACACCTTCTTCA |
| PKD1 | 16-002,087149 | CTTGCCGAGGGGGTCAGCAGCCCAGCCCCTACCCA | AGACACCCACATGGAAACGGACCTGCTCAGCAGCCTG |
| PKD2 | 04-089,176429 | ACTACACCCGGATGATGTCACAGCTCTTCCTA | GACACCCCCGTGTCCAAAACGGAGAAAACTAACTTTAAAACTCTGTC |
| PKD2 | 04-089,215013 | GAGCATTCCATCGGCAGCATAGTGTCCAA | GATTGACGCCGTGATCGTGAAGCTAGAGA |

LPO is the 5' half of the probe, RPO is the 3' half of the probe

**5. Sanger Sequencing**

Long polymerase chain reaction (PCR) was performed with KOD FX Neo (Toyobo, Tokyo, Japan) according to the manufacturer’s instructions. The reaction volume was 25 μL, which included 12.5 μL of 2×PCR buffer for KOD FX Neo, 5 μL of 2 mM dNTP mix, 2 μL (30–50 ng) of template DNA, 0.75 μL of 10 μM forward primer, 0.75 μL of 10 μM reverse primer, 0.5 μL of KOD FX Neo, and 3.5 μL of PCR-grade water. The PCR conditions were as follows: pre-denaturation at 94 °C for 2 min, 5 cycles of denaturation at 98 °C for 10 s and extension at 74 °C for 30 s per kilobase (kb), 5 cycles of denaturation at 98 °C for 10 s and extension at 72 °C for 30 s per kb, 5 cycles of denaturation at 98 °C for 10 s and extension at 70 °C for 30 s per kb, 25 cycles of denaturation at 98 °C for 10 s and extension at 68 °C for 30 s per kb, and extension at 68 °C for 7 min. Long PCR primers and sequencing primers for PKD1 are shown in Table S1 [29]. Normal PCR was performed with HotStarTaq DNA Polymerase (Qiagen, Hilden, Germany). The PCR conditions were as follows: 96 °C for 15 min, 35 cycles of denaturation at 96°C for 45 s, annealing at 57 °C for 45 s, and elongation at 72 °C for 1 min and 72 °C for 15 min. PCR primers for PKD2 and GANAB are shown in Table S2. Sanger sequencing was performed with an Applied Biosystems 3130 Genetic Analyzer (Applied Biosystems, Waltham, MA, USA). The sequence results were analyzed with the BioEdit software program and compared using the Ensembl database.

1. **Clinical outcomes and clinical follow-up**

Clinical outcome and follow-up data were collected until January 2024. Annual follow-ups involved a combination of in-person visits and electronic communications. Structured questionnaires were administered to gauge participants' reproductive status, including any pregnancies, births, miscarriages, or infertility. In addition, for couples experiencing anomalies during pregnancy who expressed interest in understanding the causes of birth defects in their offspring, we conducted more in-depth testing and analysis, including ES, Sanger sequencing, multiple ligation-dependent probe amplification (MLPA) and copy number variation sequencing (CNV-seq).

1. **Statistical analysis**

The data are presented as individual values. Statistical analysis was conducted using GraphPad

Prism 8.0 software (GraphPad Software, Inc., USA) and R (version 4.0). Descriptive analyses of the variables are expressed as the mean (standard deviation [SD]) or number (%). Continuous variable data were compared using the t test, and categorical data were compared using the χ2 test or Fisher’s exact test.

**References**

1. Bolger AM, Lohse M, Usadel B: Trimmomatic: a flexible trimmer for Illumina sequence data. *Bioinformatics* 2014, 30(15):2114-2120.

2. Li H, Durbin R: Fast and accurate short read alignment with Burrows-Wheeler transform. *Bioinformatics* 2009, 25(14):1754-1760.

3. McKenna A, Hanna M, Banks E, Sivachenko A, Cibulskis K, Kernytsky A *et al*: The Genome Analysis Toolkit: a MapReduce framework for analyzing next-generation DNA sequencing data. *Genome Res* 2010, 20(9):1297-1303.

4. Wang K, Li M, Hakonarson H: ANNOVAR: functional annotation of genetic variants from high-throughput sequencing data. *Nucleic Acids Res* 2010, 38(16):e164.

5. Li Q, Wang K: InterVar: Clinical Interpretation of Genetic Variants by the 2015 ACMG-AMP Guidelines. *Am J Hum Genet* 2017, 100(2):267-280.

6. Sim NL, Kumar P, Hu J, Henikoff S, Schneider G, Ng PC: SIFT web server: predicting effects of amino acid substitutions on proteins. *Nucleic Acids Res* 2012, 40(Web Server issue):W452-457.

7. Adzhubei IA, Schmidt S, Peshkin L, Ramensky VE, Gerasimova A, Bork P *et al*: A method and server for predicting damaging missense mutations. *Nat Methods* 2010, 7(4):248-249.

8. Schwarz JM, Cooper DN, Schuelke M, Seelow D: MutationTaster2: mutation prediction for the deep-sequencing age. *Nat Methods* 2014, 11(4):361-362.

9. Reva B, Antipin Y, Sander C: Predicting the functional impact of protein mutations: application to cancer genomics. *Nucleic Acids Res* 2011, 39(17):e118.

10. Choi Y, Chan AP: PROVEAN web server: a tool to predict the functional effect of amino acid substitutions and indels. *Bioinformatics* 2015, 31(16):2745-2747.

11. Kircher M, Witten DM, Jain P, O'Roak BJ, Cooper GM, Shendure J: A general framework for estimating the relative pathogenicity of human genetic variants. *Nat Genet* 2014, 46(3):310-315.

12. Ioannidis NM, Rothstein JH, Pejaver V, Middha S, McDonnell SK, Baheti S *et al*: REVEL: An Ensemble Method for Predicting the Pathogenicity of Rare Missense Variants. *Am J Hum Genet* 2016, 99(4):877-885.

13. Fowler A: DECoN: A Detection and Visualization Tool for Exonic Copy Number Variants. *Methods Mol Biol* 2022, 2493:77-88.
