# Supplementary material for: Exome sequencing for assessing the risk of 453 monogenic disorders in offspring: A study of 832 Chinese couples
Source: Clin Transl Med. 2024 Oct 25;14(11):e70074. doi: 10.1002/ctm2.70074 (PMC11511670; doi:10.1002/ctm2.70074)
Supplement: Supplementary file 2 — Supporting information [file CTM2-14-e70074-s002.docx]

Supplemental material (Figures) for

**Exome Sequencing for Assessing the Risk of 453 Monogenic Disorders in Offspring: A Study of 832 Chinese Couples**

Xulong Ding^1, a, *^, Miao Jiang^2, a^, Qin Hu^3, a^, Ruiqing Tong^4^, Lin Wang^1^, Jinxing Lv^4^, Ling Pan^3^, Jianquan Hou^5^, Jun He^1, 6, *^, Peng Zhou^1, *^

^1^ Center of Translational Medicine and Clinical Laboratory, The Fourth Affiliated Hospital of Soochow University, Medical Center of Soochow University, Suzhou Dushu Lake Hospital, Suzhou, Jiangsu 215123, China.

^2^ Department of Cardiology, The Fourth Affiliated Hospital of Soochow University, Medical

Center of Soochow University, Suzhou, China.

^3^ Suzhou Basecare Medical Laboratory Co., LTD, Suzhou, Jiangsu 215123, China.

^4^ Reproductive Medicine Center, The Fourth Affiliated Hospital of Soochow University, Medical Center of Soochow University, Suzhou Dushu Lake Hospital, Suzhou, Jiangsu 215123, China.

^5^ Department of Urology, The Fourth Affiliated Hospital of Soochow University, Suzhou 215000,

China.

^6^ HLA Laboratory of Jiangsu Institute of Hematology, Collaborative Innovation Center of

Hematology, The First Affiliated Hospital of Soochow University, 13/F (West), Hospital

Comprehensive Building, No.899 Ping Hai Road, Suzhou 215031, Jiangsu, China.


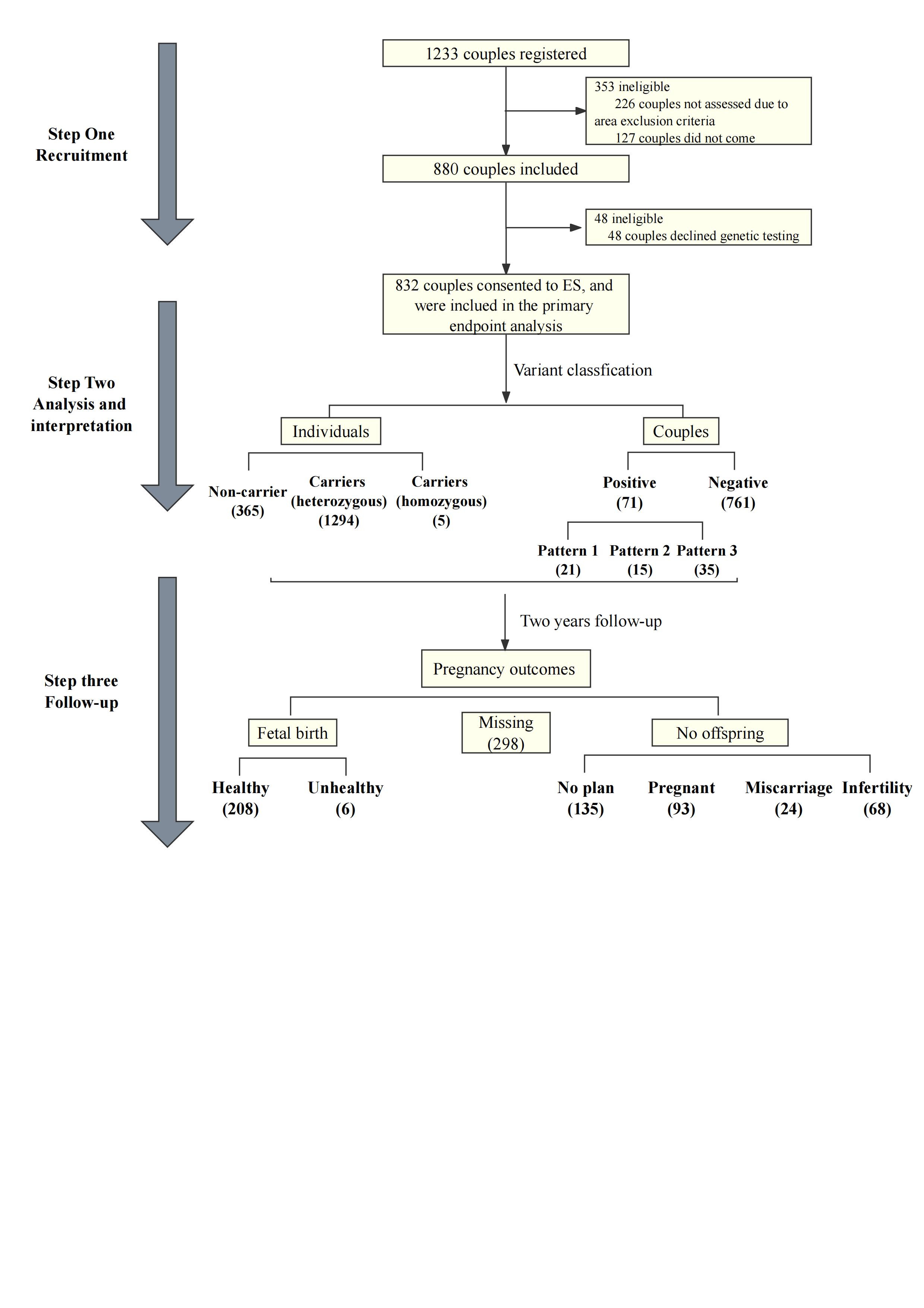


**Figure S1.** The pipeline of cohort recruitment, ES data analysis and follow-up in our study.


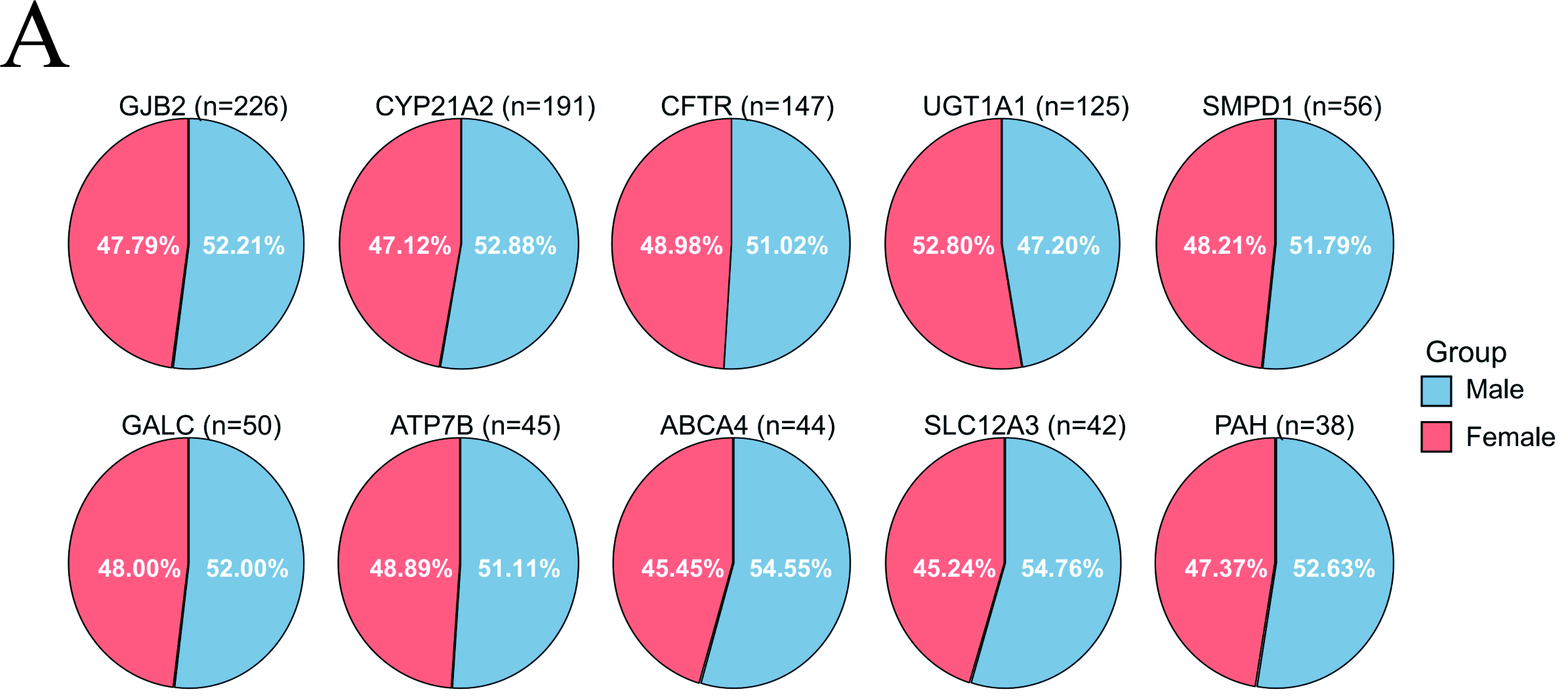


**Figure S2.** Ten genes with the highest frequency of P or LP variants among individuals.


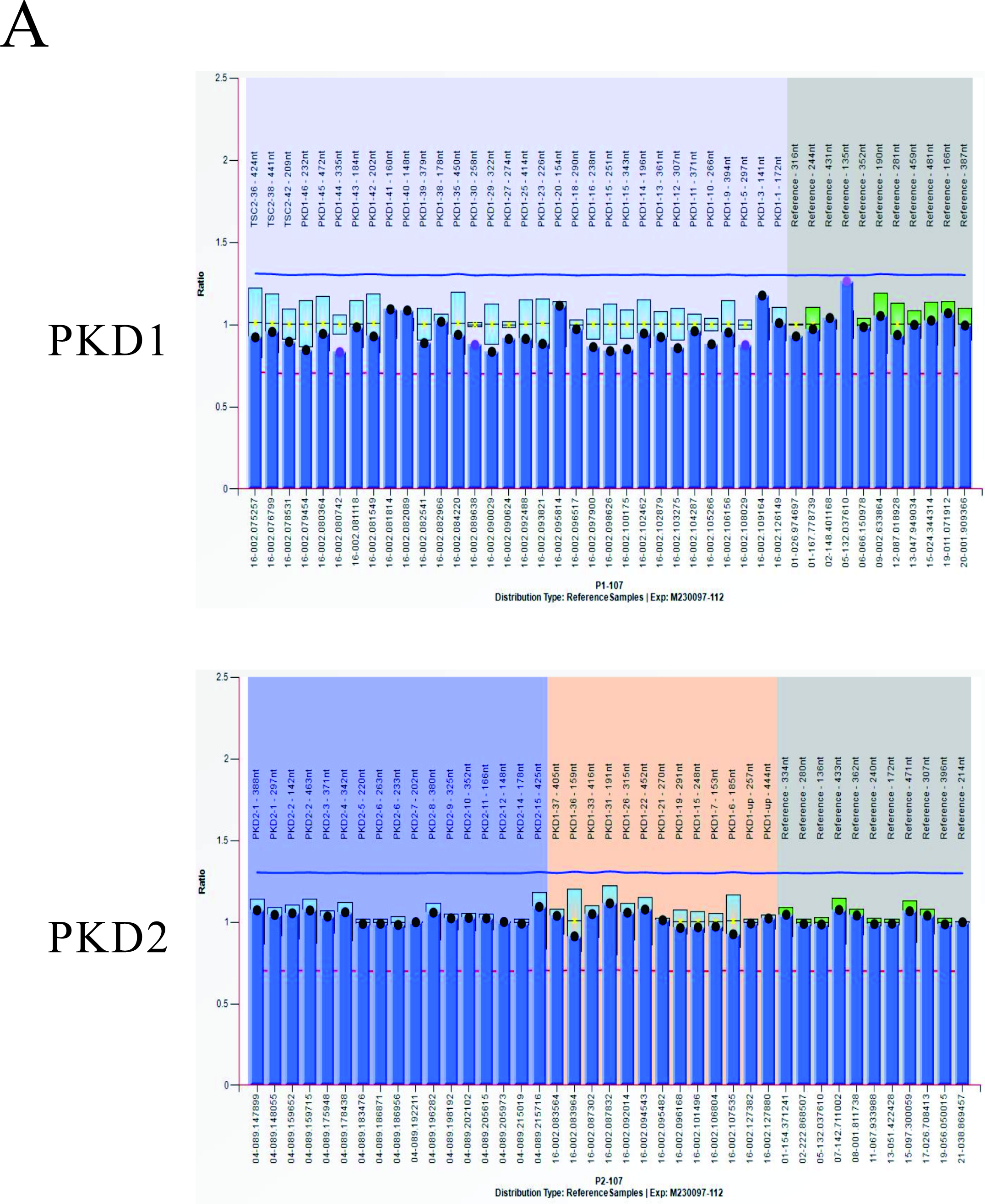


**Figure S3.** The copy number detection profile for *PKD1* and *PKD2* of the child;
